# Short-Term High-NaCl Dietary Intake Changes Leukocyte Expression of VLA-4, LFA-1, and Mac-1 Integrins in Both Healthy Humans and Sprague-Dawley Rats: A Comparative Study

**DOI:** 10.1155/2019/6715275

**Published:** 2019-09-17

**Authors:** Martina Mihalj, Anita Matić, Zrinka Mihaljević, Lidija Barić, Ana Stupin, Ines Drenjančević

**Affiliations:** ^1^Department of Physiology and Immunology, Faculty of Medicine, Josip Juraj Strossmayer University of Osijek, Osijek, Croatia; ^2^Department of Dermatology and Venereology University Hospital Osijek, Osijek, Croatia; ^3^Department of Pathophysiology, Physiology, and Immunology, Faculty of Dental Medicine and Health, Josip Juraj Strossmayer University of Osijek, Osijek, Croatia

## Abstract

This study is aimed at assessing the effects of a short-term high-salt (HS) diet on the peripheral blood leukocyte (PBL) activation status in healthy rats and young human individuals. Distribution of PBL subpopulations and surface expression of integrins were determined using flow cytometry in 36 men and women on a 7-day low-salt diet (<3.2 g salt/day) immediately followed by a 7-day HS diet (~14 g salt/day) or in Sprague-Dawley (SD) rats (*n* = 24) on a 0.4% NaCl diet (aLS group) or a 4% NaCl diet (aHS group) for 7 days. The aHS group presented with an increased frequency of granulocytes, while the frequency of lymphocytes was reduced. Although in humans HS diet reduced the expression of CD11b(act) integrin on lymphocytes, the frequency of CD11b(act)-bearing cells among all PBL subsets was increased. The aHS group of rats exhibited increased expression of total CD11b/c in granulocytes and CD3 lymphocytes. The expression of CD11a was significantly reduced in all PBL subsets from human subjects and increased in the aHS group. CD49d expression on all PBL subsets was significantly decreased in both humans and rats. In human subjects, we found reduced frequencies of intermediate monocytes accompanied by a reciprocal increase in classical monocytes. Present results suggest that a short-term HS diet can alter leukocytes' activation status and promote vascular low-grade inflammation.

## 1. Introduction

In the last few decades, hypertension has been disclosed as a key risk factor for cardiovascular (CV) impairment, and the CV diseases (including myocardial infarction, heart failure, chronic kidney disease, and stroke) are the leading cause of morbidity and mortality in modern societies across the globe [1]. It is well known that increased dietary intake of NaCl is directly proportional to the rise in blood pressure and is causal in the development of hypertension [2, 3]. Daily salt intake is still twice as high (somewhat higher) than the recommended values (<5 g/day) in almost all parts of the world [4]. Furthermore, in recent years, it became evident that such excessive salt intake affects vascular and endothelial function even in the absence of blood pressure changes [5] and endothelial dysfunction underlies all CV diseases.

Besides changes in vasoactive response and oxidative stress level, endothelial dysfunction involves increased endothelial activation resulting in the (chemo) attraction of leukocytes, their transmigration to the vascular wall, and subsequent inflammation [6]; however, it is still not clarified what kind of immune mechanisms are elicited by acute high-salt (HS) intake. It is a noteworthy fact that the increased salt intake changes the excitability of the sympathetic nervous system and that this could lead to the autonomic activation of the immune cells in the spleen and other peripheral lymphoid organs [7]. A very few studies (both animal and human) are aimed at examining the immune response to HS intake in the healthy population. Moreover, most of these studies were exclusively focused on adaptive immune responses by addressing T helper 17 (Th17) and regulatory T cell (Treg) activation. Results of the aforementioned studies imply an imbalance in Th17/Treg function induced by increased NaCl intake in the inflammation and final organ damage (endothelium dysfunction) during HS diet [8–10]. Similarly, impaired suppressive Treg function during HS load contributes to augmented Th1/Th17-mediated inflammation in autoimmune disease [11]. Interestingly, excessive salt intake has been linked to increased oxidative stress [12–14], aggravated inflammation, and pathophysiological differentiation of monocytes leading to organ damage even in treated hypertensive patients, suggesting a blood pressure-independent effect [15–18].

During inflammation, leukocytes interact with activated vascular endothelial cells, and these interactions are feasible by virtue of adhesion molecules present on the leukocyte cell surface (e.g., integrins and selectins) interacting with complementary ligands on endothelial cells [19, 20]. Common integrins expressed on leukocytes include leukocyte function-associated antigen 1 (LFA-1 or *α*L*β*2), Macrophage-1 antigen (Mac-1 or integrin *α*M*β*2), and very late antigen 4 (VLA-4 or *α*4*β*1) [21]. LFA-1 and VLA-4 recognized by the endothelial cells expressed intercellular adhesion molecules 1–5 (ICAMs 1–5) and the vascular intercellular adhesion molecule 1 (VCAM-1), respectively. Mac-1 binds to several ligands, including extracellular matrix proteins like fibrinogen and fibronectin, and activated complement components like iC3b. It has been shown that the functional state, expression, and cell-surface clustering of integrins are influenced by lipid, cytokine, and chemokine signaling molecules and by “cross-talk” from other surface adhesion molecules; however, we still lack a complete understanding on the prevailing mechanisms under various pathophysiological conditions (i.e., high salt intake) [21, 22].

Even though studies on the effect of acute salt loading on vascular reactivity in both healthy animals and the human population brought up similar results [12, 13, 23, 24], the concept that animal research (particularly in relation to pharmaceuticals and environmental agents) may be a poor predictor of human physiological responses is still present [25]. In addition, there is a lack of both animal and human studies on the effect of acute HS intake on immune system activation in otherwise healthy animals and human subjects based on the determination of leukocyte subpopulation distribution, as well as their dynamics and functional changes. The hypothesis of the present work is that high dietary salt intake could facilitate endothelial-leukocyte interactions. Thus, this study was designed as a comparative research study with the aim of assessing the effect of 7-day HS intake on (1) the frequencies of peripheral blood leukocyte (PBL) subpopulations (granulocytes, macrophages, and lymphocytes) in healthy young human subjects and Sprague-Dawley (SD) rats; (2) dynamics of leukocytes' activation by the assessment of CD11a, CD49d, activated CD11b integrin in humans, and total CD11b/c integrin in SD rats; (3) neutrophil activation in humans by the assessment of CD66b expression; and (4) distribution of the monocyte subpopulation in peripheral blood of young healthy human subjects, including classical, intermediate, and nonclassical monocytes.

## 2. Materials and Methods

### 2.1. Study Design

In order to examine similarities and differences in immune response to short-term (7 days) HS loading in healthy animal and human populations, this study was designed as comparative research that was conducted in both healthy Sprague-Dawley (SD) rats and young healthy human subjects who all underwent a similar study protocol. The animal study was an experimental (interventional) study with a randomly assigned experimental HS diet and control low-salt (LS) diet group design (the aHS diet group and the aLS diet group, respectively). The human study was an experimental (interventional) study with a nonrandomized pretest (LS diet, “washout” period)-posttest (HS diet) control group design.

### 2.2. Animal Study Design

The animal study was performed using healthy SD rats of both sexes aged 10 to 11 weeks. The animals were grown and housed at the animal care facility of the Faculty of Medicine at the Josip Juraj Strossmayer University of Osijek, Croatia, which is a nationally registered and certified user/breeder of mice and rats for educational and scientific purposes. The procuration of animals, husbandry, and all experimental procedures conformed to the European Guidelines for the Care and Use of Laboratory Animals (directive 86/6d09) and the “European Convention for the Protection of Vertebrate Animals used for Experimental and other Scientific Purposes” (Council of Europe No. 123, Strasbourg 1985). Experiments were approved by the Ethical Committee of the Faculty of Medicine, University of Osijek (Class: 602-04/14-08/06, No.: 2158-61-07-14-04) and authorized by the Ministry of Agriculture of the Republic of Croatia. All measurements were performed in the Laboratory for Vascular Physiology and Laboratory for Molecular and Clinical Immunology at the Department of Physiology and Immunology, Faculty of Medicine, Josip Juraj Strossmayer University of Osijek.

At the age of 10-11 weeks, 24 animals of both sexes were randomly divided into 2 groups and introduced into a 7-day diet protocol. The animal low-salt group (aLS group) comprised of 12 rats that were fed with standard rat chow containing 0.4% NaCl (Mucedola, Italy), while the animal HS group (aHS group) comprised of 12 rats that were fed with rat chow containing 4% NaCl (Mucedola, Italy). All animals had free access to rat chow and tap water during the whole diet protocol.

### 2.3. Animal Body Weight and Arterial Blood Pressure Measurement

Rat weight was measured at the last day of the specific dietary protocol. Blood pressure measurement was performed after a specific dietary protocol. Rats were anaesthetized with a combination of ketamine (75 mg/kg) and midazolam (2.5 mg/kg). A PE-50 catheter was inserted into the left femoral artery and mean arterial blood pressure was measured with a Spacelabs Medical monitoring system (Spacelabs Medical, Inc., Redmond, WA, USA). Animals were left for a period of 10 min for blood pressure to stabilize, followed by 1 min continuous recording of the blood pressure. Data are calculated as average arterial blood pressure from the obtained values [12].

### 2.4. Animal Blood Sample Collection and Flow Cytometry

Before measuring the blood pressure of all animals, rats were anaesthetized with a combination of ketamine (75 mg/kg; Ketanest S 25 mg/ml, Pfizer) and midazolam (2.5 mg/kg; Midazolam Torrex 5 mg/ml, Torrex Chiesi Pharma). After the blood pressure assessment, animals were decapitated and samples of mixed blood (venous and arterial) were collected in EDTA anticoagulant tubes and stored on ice to prevent cells from dying.

Prior to staining, cells (90 *μ*l of whole blood) were incubated with rat Fc block (BD Biosciences, 2 *μ*l per sample) and then, without rinsing, incubated with a mixture of antibodies comprising three different panels for 20 min at room temperature in the dark. Final staining volume was 100 *μ*l, including 8 *μ*l of antibody mixture as follows: the first panel was a mixture of CD3 PerCP-eFluor® 710 (clone: eBioG4.18, eBioscience, concentration 0.2 mg/ml, 1.25 *μ*l of antibody per sample), CD4 FITC (clone: SK3, eBioscience, concentration 6 mg/500 *μ*l, 0.5 *μ*l of antibody per sample), CD11b/c PE (clone: OX42, eBioscience, concentration 0.2 mg/ml, 0.5 *μ*l of antibody per sample), CD49d APC (clone: MRa4-1, eBioscience, concentration 0.2 mg/ml, 2 *μ*l of antibody per sample), MHC II FITC (clone: HIS19, eBioscience, concentration 0.5 mg/ml, 1.75 *μ*l of antibody per sample), and CD43 biotin (clone: W3/13, BioLegend, concentration 0.5 mg/ml, 0.5 *μ*l of antibody per sample); the second panel was a mixture of CD11b/c PE (clone: OX42, eBioscience, concentration 6 mg/500 *μ*l, 0.5 *μ*l of antibody per sample), CD49d APC (clone: MRa4-1, eBioscience, concentration 0.2 mg/ml, 2 *μ*l of antibody per sample), and granulocyte marker FITC (clone: HIS48, eBioscience, concentration 0.5 mg/ml, 0.37 *μ*l of antibody per sample); and the third panel was a mixture of CD3 PerCP-eFluor® 710 (clone: eBioG4.18, eBioscience, concentration 0.2 mg/ml, 1.25 *μ*l of per sample), CD4 FITC (clone: SK3, eBioscience concentration 6 mg/500 *μ*l, 0.5 *μ*l of antibody per sample), CD11a PE (clone: WT.1, eBioscience, concentration 0.1 mg/ml, 1 *μ*l of antibody per sample), CD49d APC (clone: MRa4-1, eBioscience, concentration 0.2 mg/ml, 2 *μ*l of antibody per sample), and CD8 PE-Cy7 (clone: OX8, eBioscience, concentration 0.2 mg/ml, 1.25 *μ*l of antibody per sample). The erythrocytes were lysed by incubating cells with 1x BD Pharm Lyse™ lysing solution for 15 minutes at room temperature, protected from light, followed by two washing steps with 1 × PBS (5 min centrifugation at 400 g and 18°C each). In the case of using a biotinylated antibody, cells were additionally labeled with 100 *μ*l streptavidin APC-Cy7 (dilution 1 : 800 with 1 × PBS, BioLegend) for 30 minutes at room temperature in the dark and then washed with 1 × PBS. After the last washing process, the cells were fixed with 1% formaldehyde. At least 10,000 target cells were collected using a BD FACSCanto II Cytometer (FACSCanto II, Becton Dickinson, San Jose, CA, USA) and analyzed using the FlowLogic software (Inivai Technologies, Menton, Australia).

### 2.5. Human Study Population

Thirty-six young healthy individuals (20 women and 16 men) were recruited by the advertisement at the Faculty of Medicine, Josip Juraj Strossmayer University of Osijek, Croatia. Exclusion criteria included a history of smoking, hypertension, coronary artery disease, diabetes, hyperlipidemia, renal impairment, and cerebrovascular and peripheral artery disease. None of the subjects were taking drugs that could affect immune function. Written informed consent was obtained from each subject. The study protocol and procedures conformed to the standards set by the latest revision of the Declaration of Helsinki and were approved by the Ethical Committee of the Faculty of Medicine, Josip Juraj Strossmayer University of Osijek (Class: 602-04/17-08/12, No.: 2158-61-07-17-42). The study protocol was performed in the Laboratory for Clinical and Sports Physiology, and flow cytometry experiments were done at the Laboratory for Molecular and Clinical Immunology at the Department of Physiology and Immunology, Faculty of Medicine, Josip Juraj Strossmayer University of Osijek.

### 2.6. Human Study Dietary Protocols

Study protocol lasted for 14 days and comprised of three visits to the Laboratory for Clinical Physiology and Physiology of Sports (1st visit when entering the study, 2nd visit after 7 days of low-salt diet protocol that was considered a “washout” period, and 3rd visit after 7 days of HS diet protocol). During the first study week, all subjects were instructed to maintain an LS diet, with an intake of less than 3.5 g of salt per day (DASH eating plan; US Department of Health and Human Services, 2006) for 7 days. After that, during the second week, all participants were subjected to the HS diet protocol for 7 days. In order to control the amount of salt taken during the HS protocol, all subjects ate around 3.5 g of salt according to the DASH diet, and the rest of the salt was supplemented in a form of a salt powder—11.7 g of salt per day for 7 days.

### 2.7. Human Arterial Blood Pressure Measurements and 24-Hour Urine and Venous Blood Sample Analysis

Body mass index (BMI) was measured at the beginning of each study visit. Blood pressure (BP), including systolic blood pressure (SBP), diastolic blood pressure (DBP), and mean arterial pressure (MAP), and heart rate (HR) were measured at the beginning of each visit after 15 min of rest in a seated position, by using an automated oscillometric sphygmomanometer (OMRON M3, OMRON Healthcare, Inc., Osaka, Japan). The final values of BP and HR were the mean of three repeated measurements.

In order to ensure that all subjects conformed to the diet protocols, they were instructed to collect 24-hour urine samples before and after each diet protocol. 24-hour urine samples were analyzed for sodium, potassium, urea, and creatinine levels. Daily salt intake based on 24-hour urinary sodium excretion was calculated using the appropriate formula: 1 − gram salt (NaCl) = 393.4 mg Na = 17.1 mmol Na. Also, at each study visit, the peripheral venous blood samples were collected and analyzed for plasma electrolytes (sodium, potassium, and calcium) and urea and creatinine level using standard laboratory methods.

### 2.8. Human Blood Sample Collection and Flow Cytometry

At each study visit, the peripheral venous blood samples were collected for flow cytometric analysis of the peripheral blood leukocyte phenotype. Blood samples were collected in tubes containing 6–10% 0.5 M EDTA and immediately processed for cell-surface staining. 100 *μ*l of whole blood was stained and incubated with a mixture of antibodies (50 *μ*l per sample) comprising three different panels for 30 min on room temperature in the dark. The final staining volume of each sample was 150 *μ*l. The first panel was a mixture of CD45-PerCP (clone: MEM-28, EXBIO, concentration 1 mg/ml, 10 *μ*l per sample), CD14 FITC (clone: 61D3, eBioscience, concentration 100 *μ*g/500 *μ*l, 5 *μ*l of antibody per sample), CD66b PE-Cy7 (clone: G10F5, eBioscience, concentration 6 *μ*g/500 *μ*l, 5 *μ*l of antibody per sample), CD11b (akt) PE (clone: CBRM1/5, eBioscience, concentration 25 *μ*g/500 *μ*l, 2.5 *μ*l of antibody per sample), and CD16 APC (clone: eBioCB16, eBioscience, concentration 6 *μ*g/500 *μ*l, 2.5 *μ*l of antibody per sample); the second panel was a mixture of CD45-PerCP (clone: MEM-28, EXBIO, concentration 1 mg/ml, 10 *μ*l of antibody per sample), CD14 FITC (clone: 61D3, eBioscience, concentration 100 *μ*g/500 *μ*l, 5 *μ*l of antibody per sample), CD11a/LFA-1 PE-Cy7 (clone: HI111, eBioscience, concentration 0.5 *μ*g/ml, 5 *μ*l of antibody per sample), and CD16 APC (clone: eBioCB16, eBioscience, concentration 6 *μ*g/500 *μ*l, 2.5 *μ*l of antibody per sample); and the third panel was comprised of CD45-PerCP (clone: MEM-28, EXBIO, concentration 1 mg/ml, 10 *μ*l of antibody per sample), CD14 FITC (clone: 61D3, eBioscience, concentration 100 *μ*g/500 *μ*l, 5 *μ*l of antibody/per sample), CD49d PE (clone: 9F10, eBioscience, concentration 12.5 *μ*g/500 *μ*l, 2.5 *μ*l of antibody/per sample), and CD16 APC (clone: eBioCB16, eBioscience, concentration 6 *μ*g/500 *μ*l, 2.5 *μ*l of antibody per sample). Erythrocytes were lysed by cell incubation with 1x BD™ FACS™ Lysing Solution for 10 min on 37°C. Visualization of the biotinylated antibody was performed by an additional staining step with streptavidin coupled with 100 *μ*l PE-Cy7 (BD Biosciences, dilution 1 : 800 with 1 × PBS) on room temperature for 30 min in the dark. After the last washing step with 1 × PBS, cells were fixated with 1% formaldehyde. At least 10,000 target cells were collected by a BD FACSCanto II cytometer (FACSCanto II, Becton Dickinson, San Jose, CA, USA) and analyzed using the FlowLogic software (Inivai Technologies, Mentone, Australia).

### 2.9. Statistical Analysis

All results are reported as the mean ± standard deviation (SD). The sample sizes for both animal and human studies were determined based on our preliminary results. The distribution of the acquired data was assessed by the Kolmogorov-Smirnov normality test. Data from the animal study were compared by Student's *t*-test or the Mann-Whitney rank-sum test in the case of normally distributed data or data that did not follow a normal distribution pattern, respectively. Data obtained from the human study were compared using a paired Student's *t*-test or the Wilcoxon rank-sum test in the case when variables were not normally distributed. The correlations between calculated daily salt intake and corresponding immunological parameters were determined by Pearson's or Spearman's correlation tests when appropriate. *p* < 0.05 was considered statistically significant. SigmaPlot, version 11.2 (Systat Software, Inc., Chicago, IL, USA) was used for statistical analysis.

## 3. Results

### 3.1. Adherence to Dietary Regime and the Effects of HS on Body Mass, Blood Pressure, and Peripheral Blood Leukocyte Subset Frequencies in Experimental Groups

Participants' characteristics are presented in Table 1. All participants were lean, and HS diet did not induce any significant change in BMI in the young healthy population (Table 1). All participants were normotensive when entering the study (SBP 118 ± 13 mmHg, DBP 74 ± 9 mmHg, and MAP 89 ± 7 mmHg). Systolic blood pressure, diastolic blood pressure, and mean arterial pressure did not change during the HS diet period compared to the LS diet period (Table 1). Furthermore, the HS diet did not induce a significant change in HR in the young healthy population (Table 1).

As expected, urinary sodium excretion and calculated daily salt intake increased significantly after a 7-day HS diet compared to the washout period (Table 1). There was no statistically significant difference in 24-hour urine urea, creatinine, and potassium levels before and after the HS diet protocol (Table 1). The serum sodium level increased following a 7-day HS diet (Table 1). There was no significant difference in other serum electrolytes (potassium, calcium) or in urea and creatinine levels measured before and after the HS diet protocol (Table 1).

Similarly, the HS diet did not affect body weight nor the mean arterial pressure of the aHS group of rats compared to the aLS group (Table 2).

While in human subjects the HS diet did not change the frequencies of the peripheral blood leukocyte subsets (Table 3), in the case of SD rats, the aHS diet group exhibited significantly increased frequencies of granulocytes (*p* = 0.004, Table 4) at the expense of the lymphocyte population whose frequencies were significantly reduced compared to the aLS diet group (*p* = 0.004, Table 4).

### 3.2. Expression of Activated CD11b Integrin in Humans and CD11b/c Integrin in SD Rats (Mac-1 Alias CR3)

**Figure 1:**
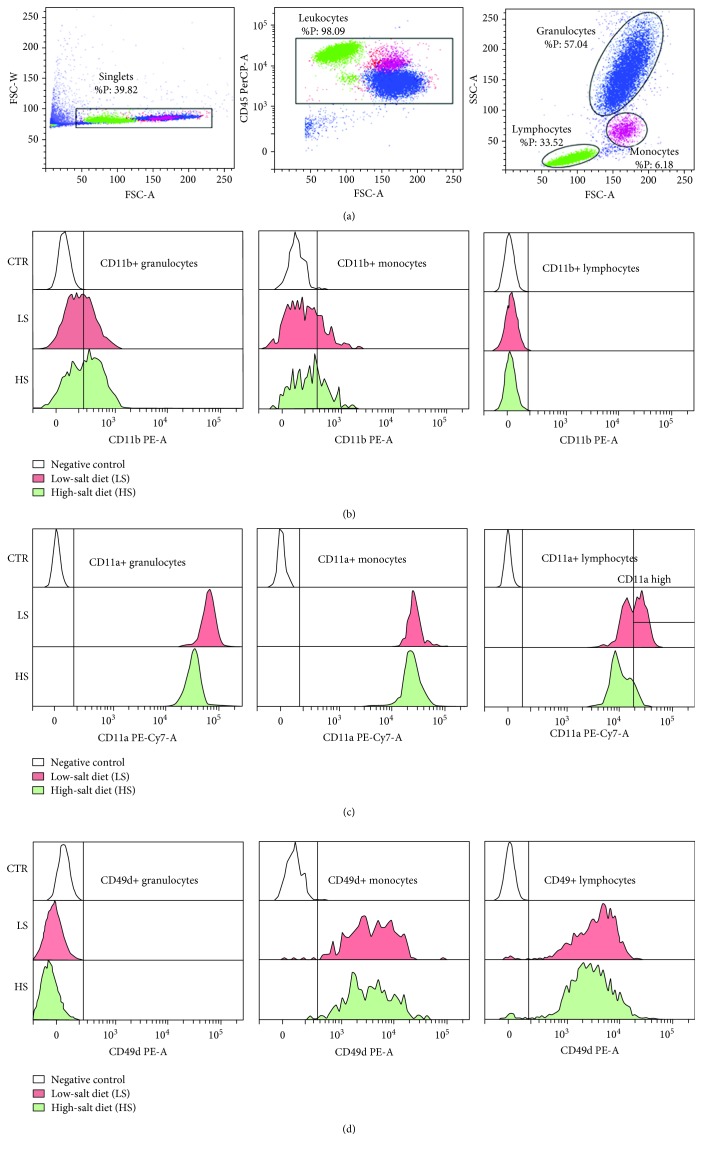
High-salt diet induced changes in integrin CD11b(act)/Mac-1, CD11a/LFA-1, and CD49d/VLA-4 expression on peripheral blood leukocyte subsets in young healthy subjects. Representative dot plots illustrating gating strategy, including exclusion of doublets using forward scatter area (FSC-A) versus forward scatter width (FSC-W) analysis; gating on CD45+ leukocytes; and gating for lymphocyte, monocyte, and granulocyte subpopulations are shown in (a). Representative histograms demonstrating integrin expression during LS and HS diets are shown for CD11b/Mac-1 (b), CD11a/LFA-1 (c), and CD49d/VLA-4 (d).

**Figure 2:**
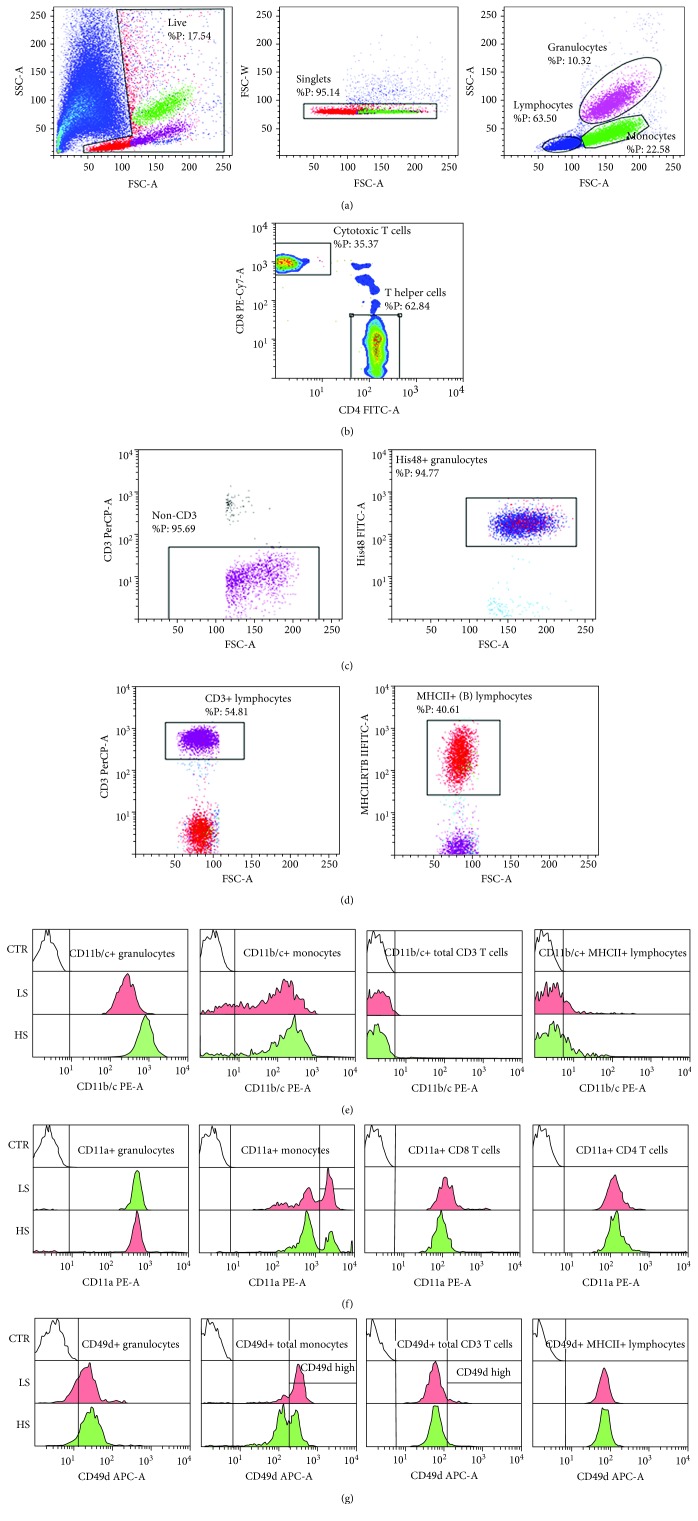
Representative histograms demonstrating integrin CD11b,c/Mac-1, CD11a/LFA-1, and CD49d/VLA-4 expression on peripheral blood leukocyte subsets in Sprague-Dawley rats. Representative dot plots illustrating gating strategy, including exclusion of doublets using forward scatter area (FSC-A) versus forward scatter width (FSC-W) analysis, and gating for lymphocyte, monocyte, and granulocyte subpopulations are shown on (a). In the case of evaluating cytotoxic T cells (CD4-CD8+) and helper T cells (CD4+CD8-), CD3+ lymphocytes were additionally analyzed for CD4 and CD8 expression (b). Granulocytes were defined on the basis of forward scatter area (FSC-A) vs. side-scatter area (SSC-A) (a), and additionally defined as CD3- and His48+ cells (c). B cells were defined as MHCII+ cells among the lymphocyte gated population (d). Representative histograms demonstrating integrin expression during LS and HS diets are shown for CD11b,c/Mac-1 (e), CD11a/LFA-1 (f), and CD49d/VLA-4 (g).

Seven days of increased salt intake induced significant changes in activated CD11b (CD11b(act)) and total CD11b/c integrin expression in healthy human subjects and SD rats, respectively (Figures 1(b) and 2(e)). Due to the limited availability of antibodies to rat leukocyte antigens, we used a monoclonal antibody to the CD11b/c epitope that is present in both the inactive and active conformations of the CD11b/c integrin allowing us to measure only the total CD11b/c expression in SD rats. As a result of this limitation, expression levels of CD11b/c were significantly higher in all leukocyte subsets in the peripheral blood of SD rats compared to human subjects (Figures 1(b) and 2(e)).

**Figure 3:**
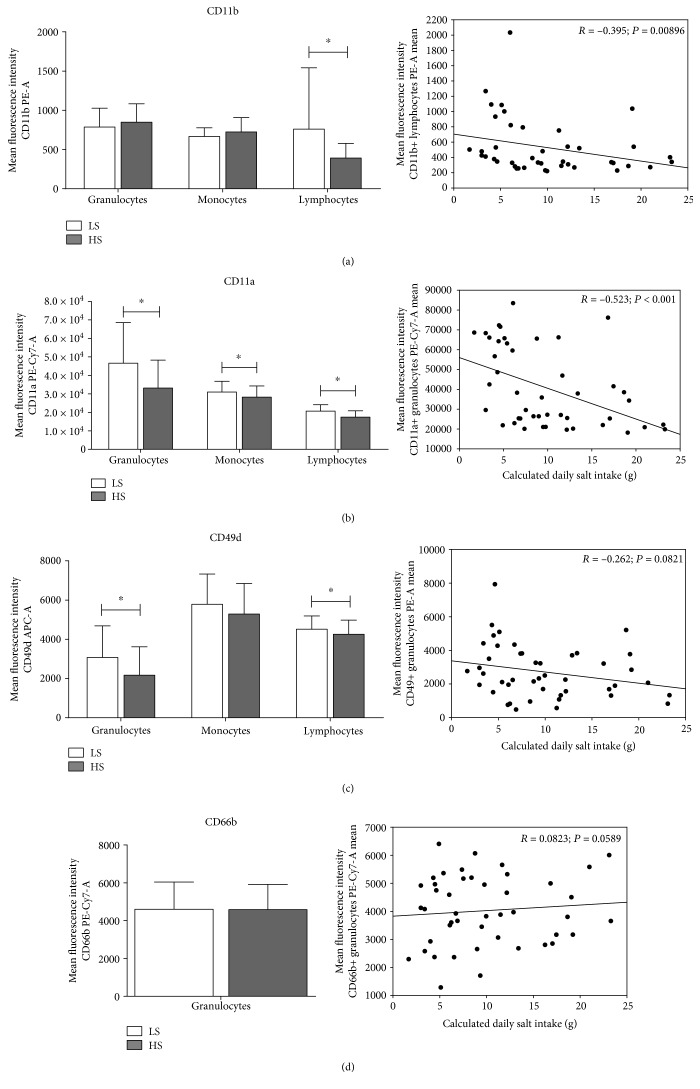
CD11b(act)/Mac-1 and CD11a/LFA-1 integrin expression on peripheral blood leukocyte subpopulations correlates to daily sodium intake in young healthy subjects during short-term dietary salt load. Average integrin expressions on peripheral blood leukocyte subsets and their correlation to daily sodium intake for CD11b/Mac-1 (a), CD11a/LFA-1 (b), CD49d/VLA-4 (c), and CD66b (c) are shown. Integrin expressions were calculated as mean fluorescence intensity of the gated cell population. Data are presented as mean ± SD and were analyzed by paired *t*-test and Pearson's correlation; *p* < 0.05 was considered significant.

Although the HS diet reduced the expression of the CD11b(act) integrin on lymphocytes (*p* = 0.009) in humans, the frequency of CD11b(act)-bearing cells among all PBL subsets was increased (Table 3, Figures 1(b) and 3(a)). Interestingly, CD11b(act) expression significantly negatively correlated to the daily salt load in humans (*r* = −0.395, *p* = 0.009; Figure 3(a)).

**Figure 4:**
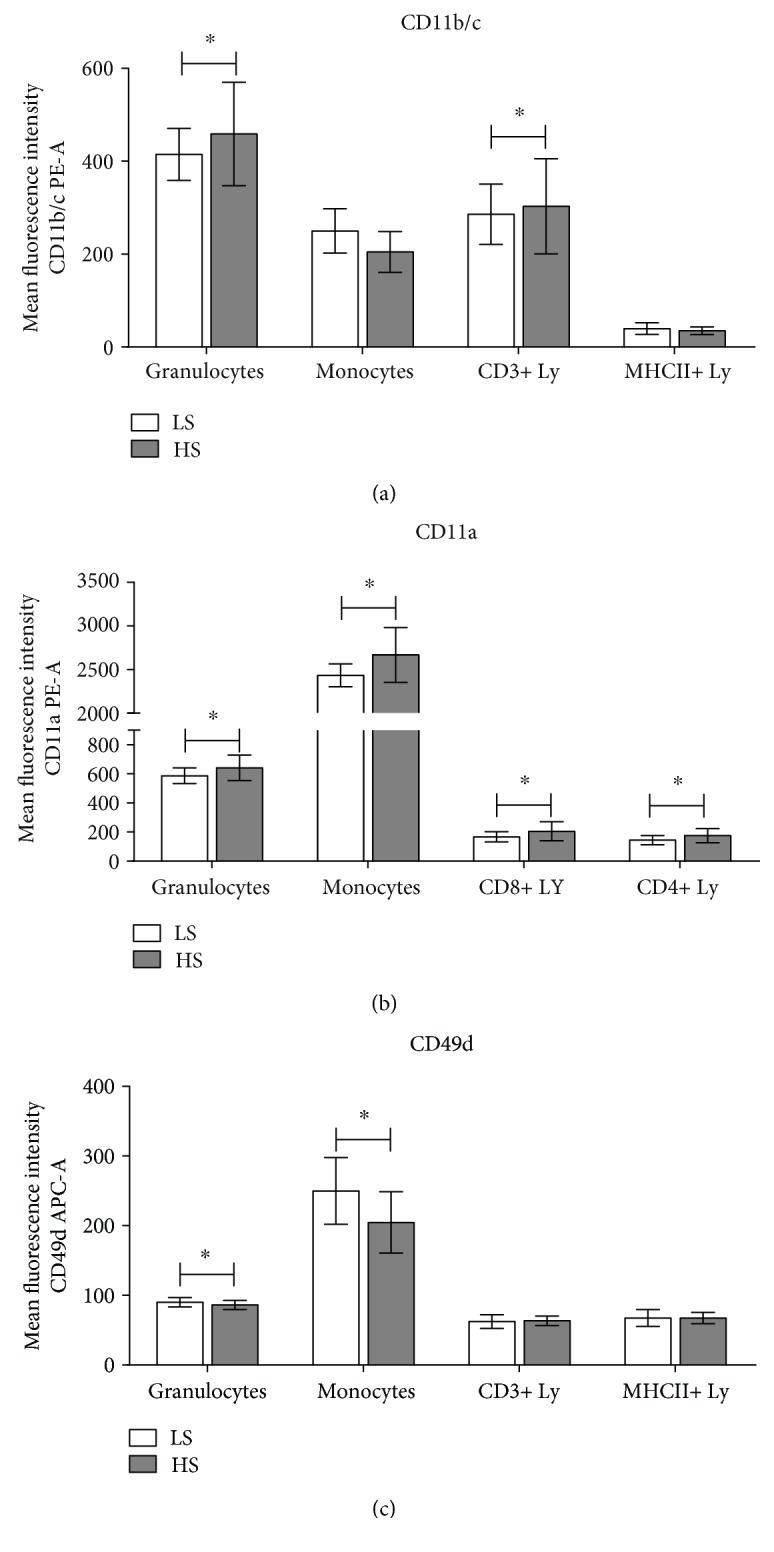
High-salt diet induced changes in integrin CD11b,c/Mac-1, CD11a/LFA-1, and CD49d/VLA-4 expression on peripheral blood leukocyte subsets in Sprague-Dawley rats. Average integrin expressions on peripheral blood leukocyte subsets for CD11b/Mac-1 (a), CD11a/LFA-1 (b), and CD49d/VLA-4 (c) are shown. Integrin expressions were calculated as mean fluorescence intensity of the gated cell population. Data are presented as mean ± SD and were analyzed by *t*-test; ^∗^*p* < 0.05 was considered significant.

In the case of SD rats, the aHS diet group exhibited a significantly increased expression of total CD11b/c in granulocytes and CD3+ lymphocytes (*p* = 0.005 and *p* = 0.049, respectively; Figures 2(e) and 4(a)). In addition, the CD11b/c-bearing lymphocyte subpopulations were more abundant in the aHS group (Table 4). Consistently, compared to lymphocyte subpopulations, monocytes and granulocytes expressed considerably higher levels of CD11b(act) and CD11b/c integrin in both healthy human subjects and SD rats, respectively.

### 3.3. Expression of CD11a (LFA-1)

In human subjects, a short-term HS diet induced a significant decrease in the CD11a expression on all leukocyte subsets from peripheral blood (Figures 1(c) and 3(b)), and this reduction significantly negatively correlated to daily salt intake in granulocytes (*r* = −0.523, *p* < 0.001; Figure 3(c)). During analysis, based on the level of CD11a expression, we have determined two lymphocyte subsets—the high and very high CD11a-expressing cells. Further analysis showed that the CD11a expression levels were significantly reduced in both subpopulations (Figure 1(c)) and that the frequency of the CD11a-very high expressing population was significantly decreased (*p* = 0.002, Table 3), while the frequency of the CD11a-high expressing population reciprocally increased during the HS diet protocol (*p* = 0.002, Table 3).

Conversely, in the case of SD rats, the HS diet resulted in significantly increased expression levels of CD11a integrin in granulocytes (*p* = 0.036, Figures 2(f) and 4(b)), monocytes (*p* = 0.015, Figures 2(f) and 4(b)), cytotoxic T cells (*p* = 0.042, Figure 4(b)), and T-helper cells (*p* = 0.035, Figures 2(f) and 4(b)). Likewise, to the human CD11a highly positive lymphocytes, the aHS group of rats presented with significantly reduced frequencies of CD11a highly positive monocytes compared to the aLS diet group (*p* = 0.015, Table 4).

### 3.4. Expression of CD49d (VLA-4)

In human subjects, a 7-day dietary salt load resulted in a significantly reduced expression of CD49d integrin on granulocytes and lymphocytes compared to the expression levels measured before entering the HS diet protocol (*p* = 0.017 and *p* = 0.009, respectively; Figures 1(d) and 3(c)). In addition, frequencies of CD49d-expressing lymphocytes were significantly reduced after the HS diet (*p* = 0.012, Table 3).

Similarly, the aHS diet group of rats exhibited a reduced CD49d expression on granulocytes and monocytes compared to the aLS diet group (*p* = 0.061 and *p* = 0.007, respectively; Figures 2(g) and 4(c)). CD49d integrin expression did not change among the CD3 T cell subsets (Figures 2(g) and 4(c)). The frequencies of the total CD49d+ monocytes were reduced after the HS diet, and further analysis revealed that there was a significant shift from the CD49d^high^-positive monocyte subset toward the CD49d^dim^-positive subset (*p* = 0.001 and *p* = 0.004, Table 4).

### 3.5. Expression of CD66b in Humans

In our study, the level of the CD66b expression on granulocytes from the peripheral blood of healthy young human subjects after the HS diet was not significantly changed (*p* = 0.553, Figure 3(d)) nor was there any significant correlation between the level of CD66b expression and daily NaCl intake (*p* = 0.589). Nevertheless, CD66b-positive granulocytes were more abundant in peripheral blood after the HS diet compared to their frequencies after the LS diet (99.4 ± 1.0 vs. 99.9 ± 0.2, *p* = 0.022), and there was a significant increase in the proportion of CD66bCD11b double-positive cells corresponding to recently activated granulocytes (63.5 vs. 70.3, *p* = 0.037).

### 3.6. Distribution of Macrophage Subsets in Human Subjects

**Figure 5:**
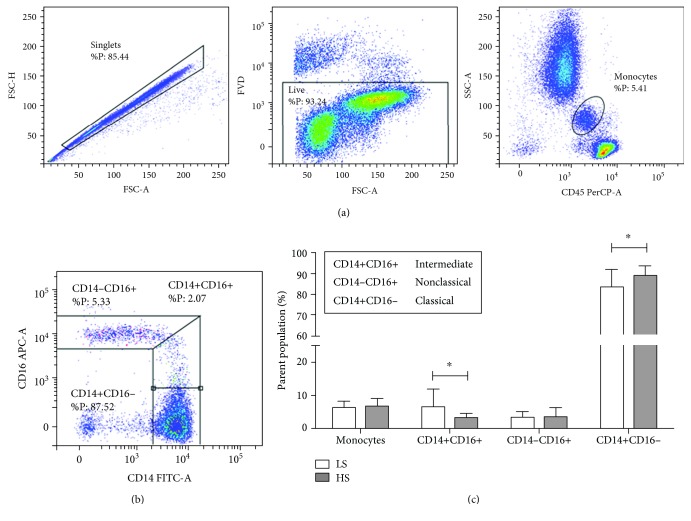
Short-term increased dietary salt load induced changes in the frequencies of classical and intermediate monocyte subpopulations in peripheral blood of healthy human subjects. (a) Representative dot plots are shown illustrating gating strategy, including exclusion of doublet cells using forward scatter area (FSC-A) versus forward scatter width (FSC-W) analysis, gating on live cells based on fixable viability dye staining, and defining monocyte population based on side-scatter area (SSC-A) versus CD45 expression. Monocyte subsets were defined based on CD14 and CD16 expression as follows: CD14-CD16+ nonclassical monocytes, CD14+CD16+ intermediate monocytes, and CD14+CD16- classical monocytes (b). Average frequencies of monocyte subpopulations are shown on bar graphs at (c). Data are presented as mean ± SD and were analyzed by *t*-test; ^∗^*p* < 0.05 was considered significant.

Seven days of increased dietary salt intake significantly changed the frequencies of peripheral blood monocyte subsets in human subjects. The frequencies of CD14+CD16+ intermediate monocytes were significantly reduced (*p* = 0.019, Figure 5(c)), whereas the frequencies of CD14+CD16- classical monocytes were increased (*p* = 0.044, Figure 5(c)) compared to the values measured immediately after the LS “washout” period.

## 4. Discussion

The salient finding of the present study is that one week of HS dietary intake has altered substantially the PBL phenotype and dynamics in both healthy SD rats and young human subjects, independently of blood pressure changes. Here, we show that (1) in SD rats, the HS dietary load resulted in a significant rise in granulocyte frequency and a reciprocal drop in the percentage of peripheral blood lymphocytes, and such effect was absent in humans. However, (2) both humans and rats on a HS diet exhibited an increased frequency of either activated CD11b or total CD11b/c-expressing cells, suggesting the recent activation of PBL. Furthermore, in humans, the HS diet reduced the expression of CD11b(act) integrin on lymphocytes and CD11a (part of LFA-1) integrin on granulocytes, both negatively correlated to daily salt intake. In the case of SD rats, increased salt intake resulted with the increased expression of total CD11b/c on granulocytes and CD3+ lymphocytes. Taken together, the expression of CD11a was significantly altered by the HS diet in rats and humans, and frequencies of some highly CD11a-positive cells were also reduced. In addition, we also found a reduced expression of CD49d on PBL subsets, both in humans and in rats on a HS diet. (3) In human subjects, the frequency of CD66b+CD11b(act)+ double-positive cells, corresponding to recently activated granulocytes, was increased during the HS diet, and (4) additional analysis of human monocyte subpopulations revealed a significant reduction in the frequencies of intermediate monocytes accompanied by a reciprocal increase in the frequency of classical monocytes.

### 4.1. Leukocyte Activation and Inflammation in the Pathogenesis of Endothelial Dysfunction during Increased Dietary Salt Intake

An increasing number of studies suggest that proinflammatory cytokines released from anchored leukocytes as well as abnormalities in leukocyte-endothelial cell interaction play an important role in the progression of cardiovascular damage in metabolic and hypertensive diseases [26–29]. Emerging evidence from studies on humans and animal models suggests that the immune system plays an important role in the target organ injury induced by high-salt loading. However, most of these studies were performed during long-term salt load or in salt-sensitive animals/humans when hypertension becomes an important etiological factor in the aforementioned processes [30–32]. On the other hand, it is important to remember that elevated blood pressure is not the sole determinant of vascular dysfunction, but changes in the renin-angiotensin system (RAS), oxidative stress level, and immune response should be taken into account as well [6, 12, 24, 33]. The rationale for the present study was the paucity of experiments, in both animal models and human subjects, investigating blood pressure-independent effects of HS loading on leukocyte-endothelial interaction and its potential role/place in the process that ultimately leads to the development of endothelial dysfunction and end organ damage (i.e., vascular function impairment).

Here, we present significant changes in the expression of several integrins (LFA-1, VLA-4, Mac-1, and CR4), as well as substantial changes in the frequencies of specific integrin-expressing cells among PBL during HS dietary load that all together suggest alterations in the PBL activation status induced by increased salt intake. In particular, recently activated Mac-1-positive cells were more abundant in both humans and rats challenged by a short-term increased dietary salt load. In addition, we found a slight increase in CD66b-bearing (activated) neutrophils in humans, which was further confirmed by the increased proportion of CD66b+CD11b(act)+ double-positive cells. This is in line with the finding of Kirabo et al. who showed that HS activates human monocytes and leads to their conversion into dendritic cells [34]. Similarly, a study on Dahl salt-sensitive rats demonstrated increased monocyte-endothelial interaction during a HS diet which could be attenuated by L-arginine administration (suggesting involvement of NO in the preservation of a healthy environment and decreased risk of leukocyte adhesion to the endothelium) [35]. Furthermore, in Dahl salt-sensitive rats, the HS diet induced increased leukocyte adhesion to the retinal microvascular endothelium through ICAM-1 linkage, leading to renal injury [36]. Importantly, the adhesion increased very quickly, just 3 days after the beginning of HS loading, when systemic blood pressure was within a normotensive range, which is in accordance with the results of the present study [36]. Earlier studies demonstrated increased leukocyte adhesion in cerebral circulation in spontaneously hypertensive, but not normotensive rats [37]. Furthermore, it has been shown that the depletion of polymorphonuclear leukocytes attenuated the development of salt-induced hypertension in Sabra hypertension-prone rats [38]. Obviously, these findings confirmed the link between the development of hypertension and inflammation. However, Takahashi et al. reported that the inhibition of ICAM-1 binding decreased leukocyte adherence but did not influence the blood pressure in Dahl S rats, and the reason for such discrepancy was not clear [36]. Importantly, the same authors reported the involvement of the RAS and increased oxidative stress level in leukocyte adhesion and the related final organ damage in Dahl S rats [36]. Similarly, we previously demonstrated increased leukocytes' reactive oxygen species production after HS intake in normotensive Sprague-Dawley rats [12]. Thus, it may be suggested that leukocyte-endothelial adhesion in response to HS loading is multifactorial, and the role of the RAS becomes apparent along with progression of vascular damage [12, 14].

### 4.2. Effects of HS Diet on the Human Peripheral Blood Monocytes

It is well accepted that monocyte/macrophage infiltration has an important role in mediating organ inflammation and its final damage. Moreover, recent studies reported that monocyte subpopulation dynamics may have prognostic values for adverse cardiovascular events [39, 40]. There are three phenotypically distinct monocyte subpopulations in the peripheral blood that also exhibit distinct pathophysiological roles, and they can be identified based on lipopolysaccharide (LPS) receptor CD14 and the Fc*γ*III receptor CD16 expression. The major function of classical monocytes (CD14+CD16-) is phagocytosis; they secrete proinflammatory cytokines such as interleukin 1 *β* (IL-1*β*), interleukin 12 (IL-12), and tumor necrosis factor (TNF) and have been found enriched in progressing atherosclerotic plaque [41, 42]. Intermediate monocytes (CD14+CD16+) display proinflammatory function and express genes involved in antigen presentation and T cell activation [42–44]. The study by Zhou et al. reported that total monocyte count remained constant during dietary HS salt intake (15 grams per day for 7 days), but intermediate monocyte population (CD14+CD16+) rapidly expanded on day 4 of the HS diet which was accompanied by a reciprocal decrease in classical and nonclassical monocyte percentages in healthy volunteers [45]. This relationship tended to be normalized during the rest of the high-salt period and was independent of changes in blood pressure levels [45]. A long-term (50 days) salt intake in healthy human subjects led to a significantly higher number of monocytes compared to the values during a lower-salt diet, indicating that dietary salt changes may lead to significant changes in immune status [46]. Furthermore, an in vitro study on the human PBL reported decreased frequencies of classical monocytes corresponding to the CD11b^+^CD14^high^CD16^low^ phenotype, accompanied by an increase in cells expressing the alternatively activated M2 macrophage phenotype (CD11b^+^CD14^low^CD16^high^) [47] and production of IL-10, TGF*β*, CCL-17, and CCR-2 anti-inflammatory cytokines with increases in *in vitro* extracellular NaCl levels. Consistent with previous reports, in the present study one week of dietary HS loading did not change total monocyte frequency in young healthy human individuals. However, unlike the aforementioned studies, we found a reduced percentage of CD14+CD16+ intermediate monocytes with a reciprocal increase in the frequencies of CD14+CD16- classical monocytes. The possible explanation for the observed discrepancies could be that we have applied a novel approach to defining gates when analyzing CD14 versus CD16 expression [48]. In addition, monocyte heterogeneity extends beyond the three subsets defined by CD14 and CD16 surface expression, and most of them have been identified in the atherosclerotic plaque, but with specific spatial- and time-dependent distribution [41, 49]. Having all that in mind, one may hypothesize that the observed changes in the frequencies of monocyte subsets represent the initiation of an inflammatory process that ultimately leads to vascular dysfunction and atherosclerosis, thus establishing HS dietary intake as an independent risk factor in the development of atherosclerosis.

### 4.3. Expression of *β*2 (LFA-1, Mac-1, and CR4) and *β*1 (VLA-4) Integrins on Peripheral Blood Leukocytes in Healthy Humans and Rats

CD11b (alias CR3 or Mac-1) is a *β*2 integrin primarily expressed on monocytes, granulocytes, macrophages, and natural killer cells; however, it has also been reported on recently activated lymphocytes [50]. This integrin mediates leukocyte adhesion and migration via specific interactions with ICAM-1, as well as phagocytosis, cell-mediated cytotoxicity, chemotaxis, and cellular activation. In the present study, an increased salt load induced significant changes in activated CD11b (CD11b(act)) and total CD11b/c integrin expression in healthy human subjects and SD rats, respectively. Due to the limited availability of antibodies to rat leukocyte antigens, we used a monoclonal antibody to a common epitope shared by CD11b (ITGAM) and CD11c (ITGAX), in their inactive and active conformation, allowing us to measure only total CD11b/c expression in SD rats. As a result of this limitation, the expression levels of CD11b/c were significantly higher in all leukocyte subsets in the peripheral blood of SD rats compared to human subjects. In human subjects challenged by a short-term dietary salt load, we found the reduced expression of CD11b(act) integrin on lymphocytes and, interestingly, increased percentage of CD11b(act)-expressing cells (corresponding to recently activated cells) among all PBL subsets suggesting their activation and transmigration to recently elicited inflammation sites. This effect could not be seen in SD rats due to the aforementioned limitations of our study design; however, increased levels of total CD11b/c in granulocytes and CD3+ lymphocytes, together with the slightly increased frequency of CD11b/c-bearing lymphocyte subpopulations, support the conclusion that the HS diet activates PBL leading to leukocyte adhesion and migration, as well other effector mechanisms involved in inflammation [51]. For example, in the model of bleomycin-induced lung fibrosis, it has been shown that the LS diet normalizes leukocyte numbers and decreases CD11b- and CD11c-expressing cells in the lungs of mice [52]. Contrary to our findings in humans, the aHS group of rats presented with increased CD11a expression in PBL, but similarly to the human study, the frequency of certain highly CD11a-positive cells was decreased, suggesting their transmigration to the sites of inflammation.

**Figure 6:**
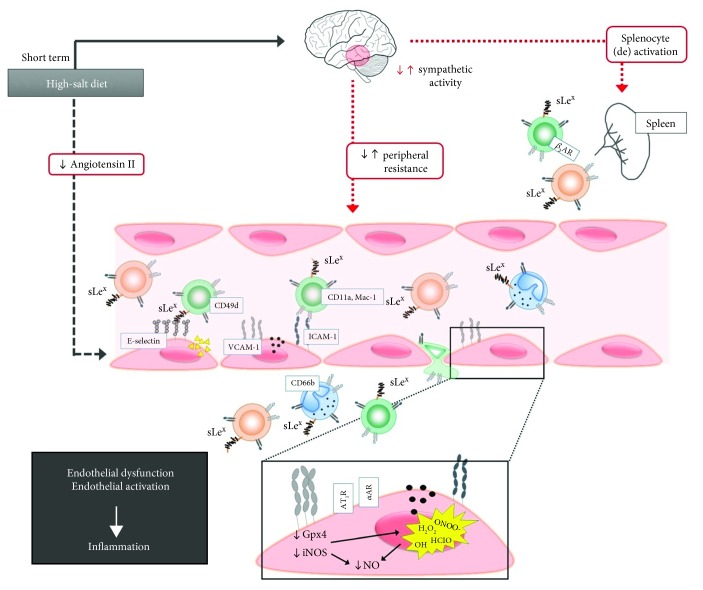
Graphical abstract. The effects of short-term excessive dietary salt intake on the immune system functions (schematic overview). This figure presents the likely effects of increased dietary salt intake on the immune system. In our previous work, in both human and animals, we have demonstrated that short-term HS diet-mediated inhibition of the RAAS leads to impaired endothelium-dependent vasodilatation due to increased oxidative stress and reduced NO bioavailability [12–14]. This could also lead to endothelial activation resulting in increased cell adhesion molecule expression (i.e., ICAM-1 and 2, VCAM-1, and E-selectin), chemokine and cytokine secretion, and thus leukocyte attraction, activation, and extravasation into the tissue [13, 53]. This is supported by the results of the present study where we found significant changes of integrin expression (LFA-1, VLA-4, and Mac-1) on the surface of peripheral blood leukocytes which may lead to their specific interaction with CAMs on activated endothelial cells. Additionally, it has been shown that increased salt intake enhances sympathetic activity and leads to an increase in peripheral vascular resistance; however, this effect could also be a source of activated leukocytes since it has been shown that increased splenic sympathetic innervation results in splenocyte activation through adrenergic beta 3 receptors [54–56].

To our knowledge, this is the first comparative study (using two different experimental models) demonstrating the effects of short-term HS loading on the changes in leukocyte integrin expression which reflect leukocyte activation, adhesion to the endothelium, and extravasation. The results of the present study demonstrate that the short-term high dietary salt intake significantly alters leukocyte surface integrin expressions in both human and SD rats; however, part of our results showed differences between the species, some of which could be explained by the differences in study designs. Together, the results of this study provide evidence that the short-term HS diet can alter the leukocytes' activation status and promote vascular low-grade inflammation. This effect of HS diet is independent on blood pressure and occurs in otherwise healthy individuals. Furthermore, the results of the present study, together with previously published data [13, 53] suggest that HS dietary intake may be an independent risk factor leading to the future development of atherosclerosis, as summarized in Figure 6 (graphical abstract).

## Figures and Tables

**Table 1:** Body mass index, blood pressure, heart rate, and biochemical parameters of the human study population.

Parameter	Before HS diet	After HS diet	*p*
*n* (F/M)	36 (20/16)		
Age (years)	20.31 ± 1.43		
BMI (kg/m^2^)	23.2 ± 2.62	23.5 ± 2.59	0.061
SBP (mmHg)	113.8 ± 11.1	116.5 ± 14.0	0.199
DBP (mmHg)	72.0 ± 7.98	70.9 ± 8.06	0.382
MAP (mmHg)	86.0 ± 7.52	85.8 ± 8.81	0.938
HR (beats per minute)	77.0 ± 15.4	75.4 ± 12.9	0.654
24 h urine volume (ml)	1541.3 ± 525.5	1459 ± 616.7	0.779
24 h urine creatinine (*μ*mol/24 h/kg)	186.2 ± 58.6	172.1 ± 52.8	0.088
24 h urine urea (mmol/dU)	286.9 ± 140.8	282.0 ± 109.4	0.301
24 h urine potassium (mmol/dU)	46.4 ± 19.1	50.1 ± 21.9	0.270
24 h urine sodium (mmol/dU)	111.6 ± 45.3	234.1 ± 92.4	**<0.001** ^∗^
Calculated daily salt intake (g/day)	6.52 ± 2.64	13.7 ± 5.40	**<0.001** ^∗^
Serum sodium (mmol/l)	136.7 ± 2.9	138.1 ± 3.0	**0.027**
Serum potassium (mmol/l)	4.08 ± 0.32	4.10 ± 0.30	0.743
Serum calcium (mmol/l)	2.42 ± 0.08	2.39 ± 0.06	0.123
Serum urea (mmol/l)	3.97 ± 1.39	4.21 ± 1.12	0.112
Serum creatinine (mmol/l)	72.4 ± 17.2	70.5 ± 16.6	0.113

Results are expressed as mean ± SD. *n*: number of subjects; BMI: body mass index; SBP: systolic blood pressure; DBP: diastolic blood pressure; MAP: mean arterial pressure; HR: heart rate. ^∗^*p* < 0.05 before vs. after HS diet.

**Table 2:** Body mass and mean arterial pressure of Sprague-Dawley rats.

Parameter	LS diet	HS diet
*n*	12	12
Age (weeks)	11	11
Body mass (g)	349.5 ± 33.96	369.8 ± 26.83
Mean arterial pressure (mmHg)	112.48 ± 2.16	107.15 ± 3.01

Results are expressed as mean ± SD. *n*: number of subjects; aLS diet: low-salt diet group; aHS diet: high-salt diet group.

**Table 3:** The effect of high-salt intake on the frequencies of cells expressing CD11b, CD11a, CD49d, and CD66b on peripheral blood leukocyte subpopulations of young healthy human subjects.

Peripheral blood leukocytes	LS diet	HS diet	*p*
Granulocytes % parent	59.9 ± 9.77	58.8 ± 7.89	0.347
CD11b+ granulocytes % parent	54.6 ± 25.2	63.0 ± 28.2	**0.018** ^∗^
CD11a+ granulocytes % parent	100.0	100.0	1.000
CD49+ granulocytes % parent	2.85 ± 2.38	3.72 ± 3.35	0.456
CD66b+ granulocytes % parent	99.4 ± 1.01	99.9 ± 0.15	**0.022** ^∗^
Monocytes % parent	5.49 ± 2.08	5.07 ± 1.88	0.287
CD11b+ monocytes % parent	19.0 ± 12.6	31.6 ± 24.5	**0.008** ^∗^
CD11a+ monocytes % parent	100.0	100.0	1.000
CD49d+ monocytes % parent	98.3 ± 1.80	98.2 ± 1.88	0.689
Lymphocytes % parent	26.2 ± 7.51	26.3 ± 7.44	0.723
CD11b+ lymphocytes % parent	0.71 ± 0.52	1.43 ± 1.67	**0.009** ^∗^
CD11a+ total lymphocytes % parent	100.0 ± 0.02	100.0 ± 0.03	0.563
CD11a very high lymphocytes % parent	59.2 ± 10.5	47.6 ± 12.5	**0.002** ^∗^
CD11a high lymphocytes % parent	40.8 ± 10.5	52.4 ± 12.5	**0.002** ^∗^
CD49d+ lymphocytes % parent	94.4 ± 13.1	93.8 ± 14.0	**0.012** ^∗^

LS: low-salt diet; HS: high-salt diet; *n* = 24 per group; data was compared using a paired *t*-test, and *p* < 0.05 was considered significant.

**Table 4:** The effect of high-salt intake on the frequencies of cells expressing CD11b/c, CD11a, and CD49d on peripheral blood leukocyte subpopulations of Sprague-Dawley rats.

Peripheral blood leukocytes	LS diet	HS diet	*p*
Granulocytes % parent	8.86 ± 2.82	14.5 ± 6.98	**0.004** ^∗^
CD11b/c+ granulocytes % parent	100.0 ± 0.01	100.0 ± 0.01	1.000
CD11a+ granulocytes % parent	97.7 ± 2.64	97.6 ± 2.14	0.858
CD49d granulocytes % parent	90.1 ± 6.74	86.3 ± 6.66	0.111
Monocytes % parent	17.5 ± 4.77	18.4 ± 3.60	0.553
Non-CD3 mononuclear cells % parent	78.6 ± 9.95	85.4 ± 8.49	**0.039** ^∗^
CD11b/c+ monocytes % parent	85.5 ± 5.38	83.2 ± 11.1	0.438
CD11a total monocytes % parent	99.5 ± 0.35	99.1 ± 0.80	0.209
CD11a^high^ monocytes % parent	53.7 ± 7.45	45.7 ± 10.4	**0.015** ^∗^
CD49d total monocytes % parent	97.3 ± 1.41	94.7 ± 4.46	**0.025** ^∗^
CD49d^high^ monocytes % parent	57.5 ± 11.1	42.3 ± 13.5	**0.001** ^∗^
CD49d^dim^ monocytes % parent	39.7 ± 11.8	52.1 ± 11.5	**0.004** ^∗^
Lymphocytes % parent	63.6 ± 5.49	55.2 ± 8.89	**0.002** ^∗^
CD3+ lymphocytes % parent	46.9 ± 5.11	45.6 ± 4.52	0.434
CD11b/c+CD3+ lymphocytes % parent	0.31 ± 0.25	0.50 ± 0.33	0.077
CD11b/c+ B cells % parent	4.89 ± 2.37	6.65 ± 3.18	0.064
CD11a+CD3+ T cells % parent	99.9 ± 0.02	99.9 ± 0.02	0.132
CD11a+ cytotoxic lymphocytes % parent	100.00	100.00	1.000
CD11a+ helper % parent	100.00	100.00	1.000
CD49d+CD3+ lymphocytes % parent	98.4 ± 1.12	96.2 ± 3.89	**0.034** ^∗^
CD49d+CD3+ high lymphocytes % parent	5.45 ± 2.38	7.42 ± 1.82	**0.011** ^∗^
CD49d+ cytotoxic lymphocytes % parent	99.9 ± 0.07	99.8 ± 0.15	0.071
CD49d+ T helper % parent	98.6 ± 0.72	98.1 ± 1.16	0.137
CD49d+ B cells % parent	99.9 ± 0.04	94.9 ± 7.43	**0.008** ^∗^

LS: low-salt diet; HS: high-salt diet; *n* = 24 per group; data was compared using *t*-test, and *p* < 0.05 was considered significant.

## Data Availability

This data availability statement refers to manuscript 6715275 titled “Short-Term High-NaCl Dietary Intake Changes Leukocyte Expression of VLA-4, LFA-1, and Mac-1 Integrins in Both Healthy Humans and Sprague-Dawley Rats: A Comparative Study” submitted to Mediators of Inflammation. With this statement, we confirm that all data used to support the findings of this study are included within the article.

## Abbreviations

aHS group: Animal high-salt group
aLS group: Animal low-salt group
BMI: Body mass index
BP: Blood pressure
CV: Cardiovascular
DBP: Diastolic blood pressure
HR: Heart rate
HS: High salt
IL-12: Interleukin 12
IL-1*β*: Interleukin 1 *β*
ICAM-1: Intercellular adhesion molecule 1
ICAM-2: Intercellular adhesion molecule 2
LFA-1: Lymphocyte function-associated antigen 1
LPS: Lipopolysaccharide
LS: Low salt
Mac-1: Macrophage-1 antigen
MAP: Mean arterial pressure
PBL: Peripheral blood leukocyte
RAS: Renin-angiotensin system
SBP: Systolic blood pressure
SD rats: Sprague-Dawley rats
TNF: Tumor necrosis factor
VCAM-1: Vascular intercellular adhesion molecule 1
VLA-4: Very late antigen 4.
